# Long-term persistence of gastric dysbiosis after eradication of *Helicobacter pylori* in patients who underwent endoscopic submucosal dissection for early gastric cancer

**DOI:** 10.1007/s10120-020-01141-w

**Published:** 2020-11-17

**Authors:** Toshio Watanabe, Yuji Nadatani, Wataru Suda, Akira Higashimori, Koji Otani, Shusei Fukunaga, Shuhei Hosomi, Fumio Tanaka, Yasuaki Nagami, Koichi Taira, Tetsuya Tanigawa, Geicho Nakatsu, Masahira Hattori, Yasuhiro Fujiwara

**Affiliations:** 1 Department of Gastroenterology, Osaka City University Graduate School of Medicine, 1-4-3 Asahi-machi Abeno-ku, Osaka, Japan; 2 RIKEN Center for Integrative Medical Sciences Laboratory for Microbiome Sciences, Yokohama, Kanagawa Japan; 3 Department of Gastroenterology, Osaka City Juso Hospital, Osaka, Japan; 4 Department of Immunology and Infectious Diseases/Genetics and Complex Diseases, Harvard T. H. Chan School of Public Health, Boston, MA USA; 5 Graduate School of Advanced Science and Engineering, Waseda University, Tokyo, Japan

**Keywords:** Dysbiosis, Gastric cancer, *Helicobacter pylori*, Eradication

## Abstract

**Background:**

Gastric microbiome, other than *Helicobacter pylori*, plays a role in the tumorigenesis of gastric cancer (GC). Patients who undergo endoscopic submucosal dissection for early GC have a high risk of developing metachronous GC even after successful eradication of *H. pylori*. Thus, we investigated the microbial profiles and associated changes in such patients after the eradication of *H. pylori*.

**Methods:**

A total of 19 *H. pylori*-infected patients with early GC who were or to be treated by endoscopic resection, with paired biopsy samples at pre- and post-eradication therapy, were retrospectively enrolled. Ten *H. pylori*-negative patients were enrolled as controls. Biopsy samples were analyzed using 16S rRNA sequencing.

**Results:**

*H. pylori*-positive patients exhibited low richness and evenness of bacteria with the deletion of several genera, including *Blautia*, *Ralstonia*, *Faecalibacterium*, *Methylobacterium*, and *Megamonas*. *H. pylori* eradication partially restored microbial diversity, as assessed during a median follow-up at 13 months after eradication therapy. However, post-eradication patients had less diversity than that in the controls and possessed a lower abundance of the five genera mentioned above. The eradication of *H. pylori* also altered the bacterial composition, but not to the same extent as that in controls. The microbial communities could be clustered into three separate groups: *H. pylori*-negative, pre-eradication, and post-eradication.

**Conclusion:**

Changes in dysbiosis may persist long after the eradication of *H. pylori* in patients with a history of GC. Dysbiosis may be involved in the development of both primary and metachronous GC after the eradication of *H. pylori* in such patients.

**Electronic supplementary material:**

The online version of this article (10.1007/s10120-020-01141-w) contains supplementary material, which is available to authorized users.

## Introduction

*Helicobacter pylori* was classified by the World Health Organization as a type I carcinogen in 1994 [1, 2]. Approximately, 89% of non-cardia gastric cancers (GCs), accounting for 78% of total GCs, are attributable to *H. pylori* infection [3]. Several randomized controlled trials have demonstrated a reduction in the risk of developing primary and secondary GC after the removal of *H. pylori* [4–6]. A meta-analysis showed that the pooled ratio for the incidence of GC in individuals who underwent eradication therapy was 0.54 as compared to that in patients who were not treated for *H. pylori* [7]. These confirmed the preventive effect of *H. pylori* eradication on GC development. However, this effect is limited and some patients develop GC after the eradication of *H. pylori*. Several factors, such as endoscopic severe atrophy, histological intestinal metaplasia, and aberrant DNA methylation, in the gastric tissues, are associated with GC in *H. pylori*-eradicated patients [8–10]; however, their underlying mechanisms are unclear.

Animal studies using hypergastrinemic insulin–gastrin (INS-GAS) transgenic mice indicated that non-*H. pylori* microbiota play a crucial role in gastric carcinogenesis. INS-GAS-mice spontaneously developed atrophic gastritis, intestinal metaplasia, dysplasia, and GC [11], whereas germ-free INS-GAS mice had minimal to no gastric pathologies and did not develop GC. Monoassociation of *H. pylori* in germ-free INS-GAS mice accelerates gastritis and induces GC, but the severity of gastric lesions in these mice is less, and there is a delayed onset of GC compared to that in *H. pylori*-infected specific pathogen-free INS-GAS mice [12, 13]. Recently, 16S rRNA-based metagenomic analysis revealed alterations in the composition and diversity of gastric microbiota in patients with *H. pylori* [14, 15]. Thus, dysbiosis may play an important role in gastric carcinogenesis.

Metachronous GC develops after the successful eradication of *H. pylori* with high incidence in patients after endoscopic submucosal dissection (ESD) for primary gastric neoplasms [6, 16]. Thus, high-risk patients are suitable candidates for analysis of the pathogenesis of GC after the eradication of *H. pylori*. In this study, we investigated the alteration in microbiota upon the eradication of *H. pylori* in the gastric mucosa using (before and after eradication) paired samples from patients who underwent ESD for early GC.

## Methods

### Patients

The study cohort comprised *H. pylori*-infected patients diagnosed with early GC who planned to undergo ESD or were being followed up after ESD at the Osaka City University Hospital and underwent baseline endoscopy between June 2013 and December 2015. We retrospectively enrolled 21 patients with paired frozen biopsy samples during baseline and follow-up endoscopy (6 months or later) after the eradication of *H. pylori*. Patients who did not have gastric neoplasms or gastroduodenal ulcers (confirmed using endoscopy) were also included. *H. pylori-naïve* patients constituted the *H. pylori*-negative control group. The study had the following exclusion criteria during patient selection: prior documented treatment for *H. pylori*; use of antibiotics, proton pump inhibitors (PPIs), probiotics, or immunosuppressive drugs within 12 weeks of endoscopy; and coexistence of concomitant illnesses, including active malignant diseases except for GC, severe renal or hepatic dysfunction, and previous abdominal surgery. The study protocols were approved by the ethics committee of the Osaka City University Graduate School of Medicine (Approval No. 2413). Written informed consent was obtained from all participants.

### Determination of *H. pylori* status

*H. pylori* infection was determined using at least one of the three tests (serum antibody, histology, and ^13^C-urea breath test [UBT]). Successful eradication was concluded with negative readouts for histology and UBT ≥ 4 weeks after the end of anti-*H. pylori* treatment. A subject was considered *H. pylori*-naïve if all of the following five criteria were met: (1) no history of *H. pylori* eradication; (2) seronegativity for *H. pylori* antibody; (3) negative UBT; (4) no pathological findings of gastric atrophy and intestinal metaplasia in biopsy specimens; and (5) none or mild gastric atrophy, as per the Kimura–Takemoto classification [17].

### *H. pylori* eradication therapies

Post-ESD patients were administered *H. pylori* eradication therapies within 4 weeks of the baseline endoscopy, while patients with newly diagnosed gastric neoplasms underwent therapy within 12 weeks of ESD. *H. pylori*-positive individuals were administered first-line triple therapy consisting of a PPI (esomeprazole, lansoprazole, or rabeprazole) or vonoprazan in combination with amoxicillin plus clarithromycin (twice daily for 7 days). Patients who did not respond to the first-line therapy were administered second-line triple therapy where clarithromycin was replaced with metronidazole.

### Sample collection

Biopsy specimens were obtained from all the patients from the greater curvature of the antrum and corpus of the stomach during baseline endoscopy. Gastric biopsy specimens were additionally obtained from *H. pylori*-positive subjects who underwent ESD at least once after 6 months of successful eradication of *H. pylori* and were subjected to microbiome analysis. The latest specimens were analyzed for eradicated patients subjected to multiple biopsies.

### DNA extraction and 16S rRNA gene amplicons sequencing

DNA was enzymatically extracted from biopsies of gastric mucosa as previously described [18]. The V1–V2 region of the 16S rRNA was amplified using polymerase chain reaction (PCR) with the forward primer 27Fmod (5′- AATGATACGGCGACCACCGAGATCTACACxxxxxxxxACACTCTTTCCCTACACGACGCTCTTCCGATCTagrgtttgatymtggctcag-3′), containing the Illumina Nextera Adapters sequcence and a unique 8-bp barcode sequence for each sample (Barcode sequence by x), and the reverse primer 338 R (5′-CAAGCAGAAGACGGCATACGAGATxxxxxxxxGTGACTGGAGTTCAGACGTGTGCTCTTCCGATCTtgctgcctcccgtaggagt-3′) containing the Illumina Nextera Adapters sequence using a 9700 PCR System (Life Technologies, Tokyo, Japan) as previously reported [19]. PCR amplicons were purified using AMPure XP magnetic purification beads (Beckman Coulter, Brea, CA, USA) and quantified using the Quant-iT PicoGreen dsDNA Assay Kit (Life Technologies Japan). An equal amount of each PCR amplicon was mixed and subjected to sequencing with MiSeq (Illumina) using MiSeq Reagent Kit v2 (500 cycles) according to the manufacturer’s instructions [20].

### Processing of 16S sequence data

We analyzed the 16S sequencing data using our previously reported method [20] with some modifications. Briefly, after demultiplexing the 16S sequence reads, paired-end reads were joined using the fastq-join program. Reads with an average quality value of < 25 and inexact matches to both universal primers were removed. Among the filter-passed reads, 3000 reads/sample were randomly chosen and analyzed. Subsequently, we sorted the selected reads using the average quality value and grouped them into operational taxonomic units (OTUs) using the UCLUST algorithm with a 97% identity threshold. To reduce the effects of sequence error, a representative sequence of the OTUs was determined to be one having the highest redundancy in the OTU. Each OTU was aligned against the publicly available 16S (RDP and CORE) and NCBI genome databases using GLSEARCH. We used sequence similarity thresholds of 70%, 94%, and 97% to assign the phylum, genus, and species, respectively.

### Statistical analysis

Details of the statistical analyses are described in the Supplementary Methods (Supplemental Text).

## Results

### Baseline patient characteristics

Among the 21 *H. pylori*-positive patients with early GC, 15 were followed up after successful ESD. The remaining six patients were scheduled to undergo ESD at enrollment; subsequently, curative ESD was performed in all six patients. All of 21 GCs were intestinal type. In 17 of the 21 patients, *H. pylori* was eradicated using first-line triple therapy, while four patients were subjected to second-line triple therapy to eradicate *H. pylori*. Gastric biopsy specimens after *H. pylori* eradication could not be obtained from one patient, and one patient was given a probiotic after the eradication of *H. pylori*. Therefore, these two patients were excluded from the study. We enrolled 13 *H. pylori*-negative (*H. pylori-naïve*) patients, but three were excluded from the study due to the use of a PPI. Table 1 shows the clinical characteristics of patients in the *H. pylori*-negative group and pre-eradication group (*H. pylori*-positive patients with early GC). There was no difference in the age and sex ratio of the patients between the two groups. The *H. pylori*-positive subjects had more severe endoscopic atrophy than that observed in the *H. pylori*-negative subjects.

### Effect of *H. pylori* infection on gastric bacterial diversity and richness

We obtained paired (before and after *H. pylori* eradication) samples from the antrum and corpus from 18 out of 19 *H. pylori*-positive patients with early GC, while in one patient, paired samples were obtained from the antrum only. Samples were obtained from both sites for eight out of the 10 *H. pylori-negative* patients, while one sample from either antrum or corpus was obtained from two patients. We obtained a total of 2,261,850 high-quality 16S reads using the Illumina MiSeq platform (Supplemental Table 1). The Good’s coverage indices of the 3000 reads per sample were 0.954 (± 0.002) and 0.934 (± 0.005) for total and *H. pylori*-removing analysis, respectively.

**Fig. 1:**
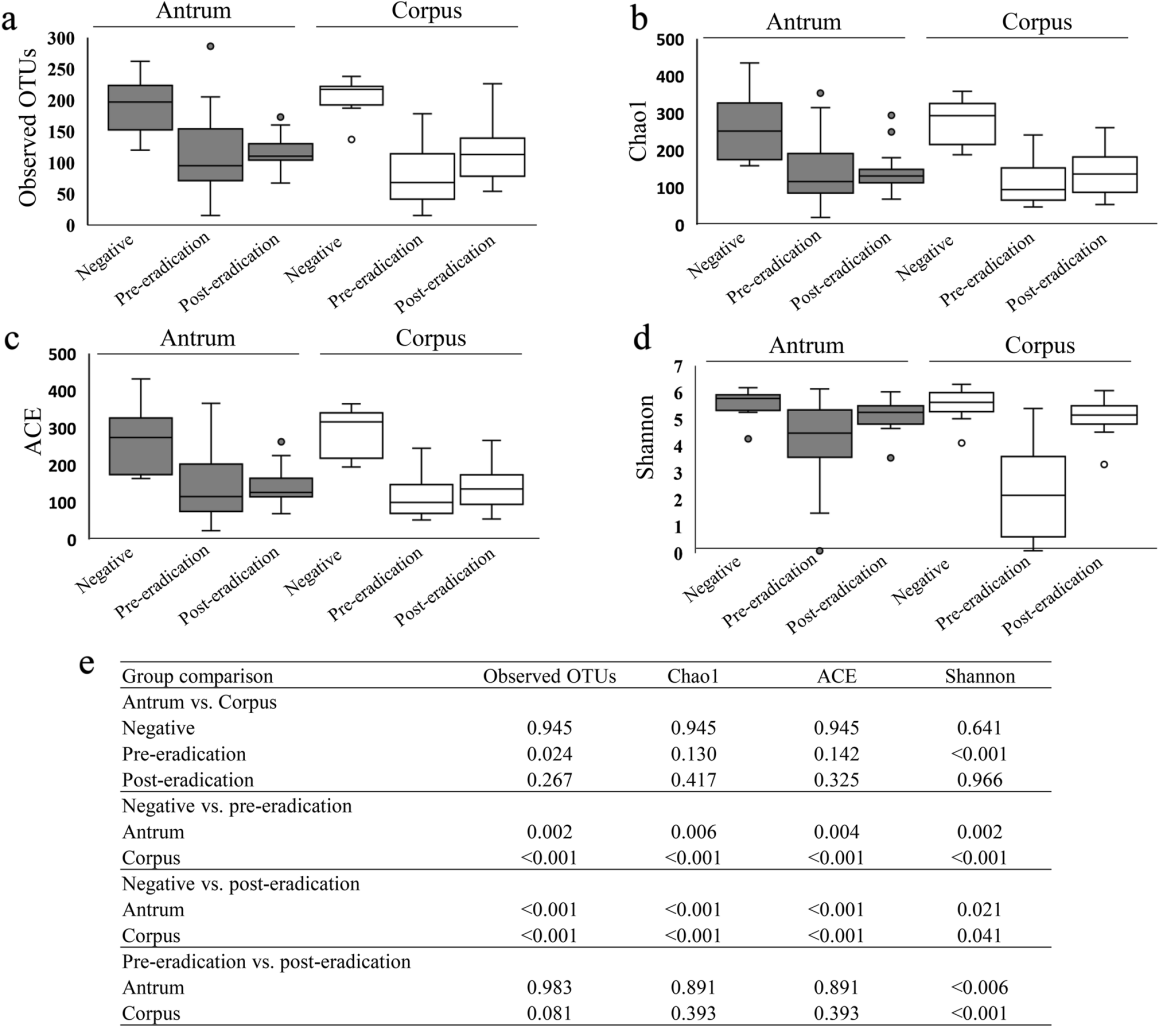
Comparison of α-diversity in the gastric microbiome between different gastric regions or *H. pylori* status. The numbers of observed OTUs (**a**) and indices of Chao1 (**b**), ACE (**c**), and Shannon (**d**) in each group have been expressed as medians and interquartile ranges. **e** The *P* values between different groups are shown. Wilcoxon rank sum test was used to compare the values of *H. pylori*-negative patients with those of patients in the pre-eradication or post-eradication groups. The Wilcoxon signed-rank test was used to compare the difference in the paired samples from the pre- and post-*H. pylori* eradication groups

**Fig. 2:**
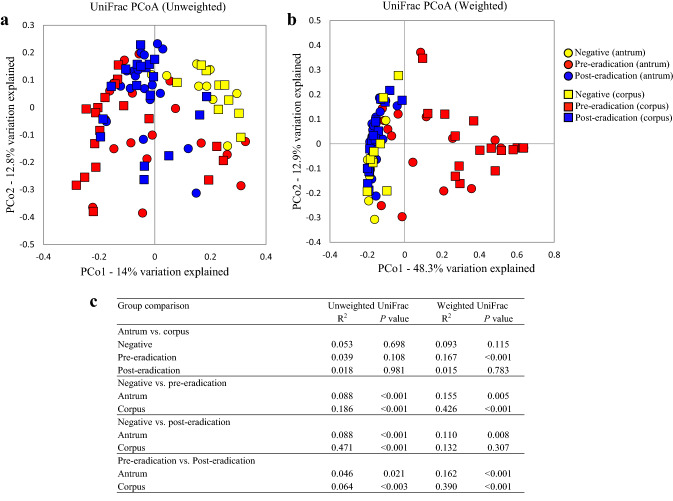
β-Diversity distances between different *H. pylori* groups using principal coordinate analysis (PCoA). **a** Unweighted UniFrac PCoA. **b** Weighted UniFrac PCoA. **c** PERMANOVA data comparing β-diversity of gastric bacterial communities between different *H. pylori* status groups using unweighted and weighted UniFrac distances

**Fig. 3:**
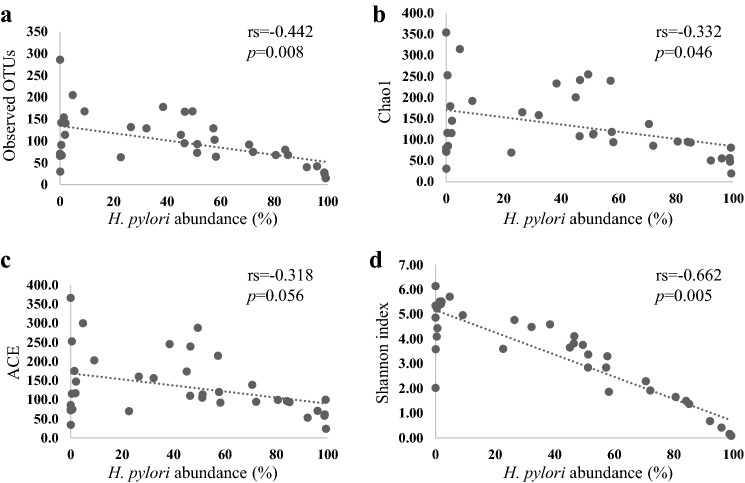
Correlation between the abundance of *H. pylori* and α-diversity of the gastric microbiome. Correlation between the abundance of *H. pylori* and the number of observed OTUs (**a**), Chao1 index (**b**), ACE index (**c**), and Shannon index (**d**). Correlation was determined using Spearman's rank method

First, we evaluated the differences in the composition of microbiota between the antrum and corpus using paired samples. In the *H. pylori*-negative group, there were no differences in the α-diversity indices, including the number of observed OTUs and Chao1, ACE, and Shannon, between these two regions (Fig. 1). Similarly, β-diversity analysis using unweighted and weighted UniFrac data showed that the bacterial communities did not differ between the regions (Fig. 2). However, in *H. pylori*-positive patients, the number of observed OTUs and the Shannon index in the corpus was significantly lower than that in the antrum, with a non-significant decrease in the indices of Chao1 and ACE in the corpus (Fig. 1). The relative abundance of *H. pylori* inversely correlated with the α-diversity indices (Fig. 3). Additionally, the weighted UniFrac analysis revealed a significant difference in the gastric microbiota structure between the antrum and corpus in the pre-eradication patients (Fig. 2).

Next, we evaluated the changes in the gastric microbiota caused by *H. pylori* infection. Compared to the *H. pylori*-negative group, the α-diversity of gastric microbiota was reduced in the pre-eradication group, as indicated by a significant reduction in the numbers of observed OTUs and Chao1, ACE, and Shannon indices in the antrum and corpus (Fig. 1). Unweighted and weighted UniFrac analyses showed that the gastric microbial community in the *H. pylori*-positive patients clustered separately from that in the *H. pylori*-negative patients (Fig. 2).

### Effect of *H. pylori* eradication on the gastric microbiota

Paired gastric samples from 19 patients with early GC were obtained after a median period of 13 months [range 8–24 months; < 12 months (*n* = 3), 12–24 months (*n* = 10), > 24 months (*n* = 6)] after successful eradication therapy. We detected *H. pylori* reads in six out of 19 antrum biopsy samples and eight out of 18 corpus biopsy samples obtained after eradication using 16S sequencing (Supplemental Table 1). However, the relative abundances of *H. pylori* in these samples were low (0.033–0.33%). Previous studies have suggested a ~ 1% cut-off for the positive readout for *H. pylori* colonization using pyrosequencing [21]. Taken together with the negative readout for histology and UBT for *H. pylori*-positive subjects after eradication therapy, we concluded that *H. pylori* was successfully eradicated in these patients.

*H. pylori* eradication significantly increased the Shannon index, but did not affect other α-diversity metrics (Fig. 1, Supplemental Figure 1). Moreover, all metrics associated with α-diversity in the antrum and corpus of patients in the post-eradication group were lower than those in the *H. pylori*-negative patients (Fig. 1). UniFrac analysis showed that the composition of the gastric bacterial community significantly changed after *H. pylori* eradication, but that in the post-eradication group did not return to normal as in the negative group. The gastric bacterial communities were clustered into three groups based on *H. pylori* status (Fig. 2).

### α- and β-diversity analysis by removing *H. pylori* reads

Since the gastric microbial community in *H. pylori*-positive subjects was dominated by *H. pylori* (Supplemental Table 2), the difference in the composition of gastric microbiota between the negative and positive groups was not surprising. Therefore, we removed *H. pylori* reads from our sample data and reanalyzed the data. Most differences in the α- and β-diversity metrics of the gastric microbiota between *H. pylori*-negative and pre-eradication groups remained robust even after removing *H. pylori* reads (Supplemental Figure 2). In contrast, the difference in the α-diversity metrics and dissimilarities in UniFrac analyses of the bacterial communities between pre- and post-eradication groups disappeared after omitting the *H. pylori* reads, except for the Shannon index and difference in weighted UniFrac in the corpus (Supplemental Figures 3, 4).

### Comparison of the microbiota composition between different *H. pylori* status groups

**Fig. 4:**
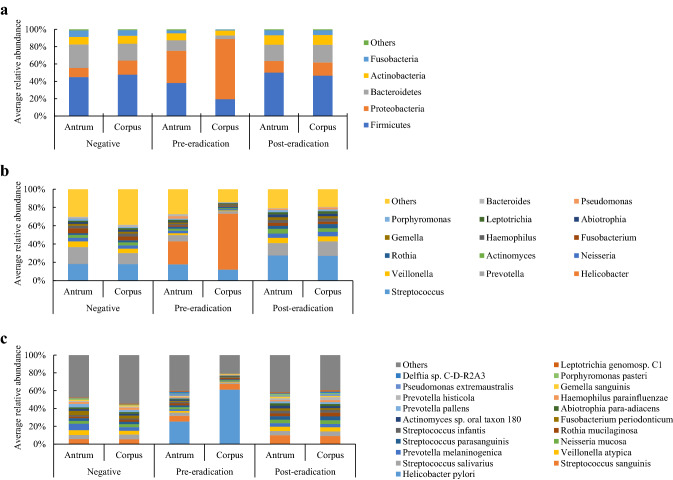
Average relative abundances of taxa in the stomach of *H. pylori*-negative, pre-eradication, and post-eradication patients. Changes in the phylum (**a**), genus (**b**), and species (**c**)

All the three groups of patients predominantly possessed the following phyla as part of their gastric microbiota: *Firmicutes*, *Proteobacteria*, *Bacteroidetes*, *Actinobacteria*, and *Fusobacteria*. Regardless of the presence of *H. pylori*, the antrum and corpus showed an abundance of *Firmicutes*, except for the corpus of the pre-eradication group wherein *Proteobacteria* (including *H. pylori*) was predominant (Fig. 4, Supplemental Table 3). Among the phyla, *Proteobacteria* was significantly enriched in the corpus of *H. pylori*-positive patients as compared to that in *H. pylori*-negative subjects, whereas the abundance of *Bacteroidetes* in the corpus and *Firmicutes, Bacteroidetes,* and *Spirochetes* in the antrum were significantly reduced (Supplemental Table 4). The eradication of *H. pylori* reduced the abundance of *Proteobacteria* and increased the abundance of *Firmicutes*, *Bacteroidetes,* and *Fusobacteria* in the gastric corpus. We found no significant differences in the relative abundance of bacteria in each phylum between the *H. pylori*-negative and post-eradication groups.

**Fig. 5:**
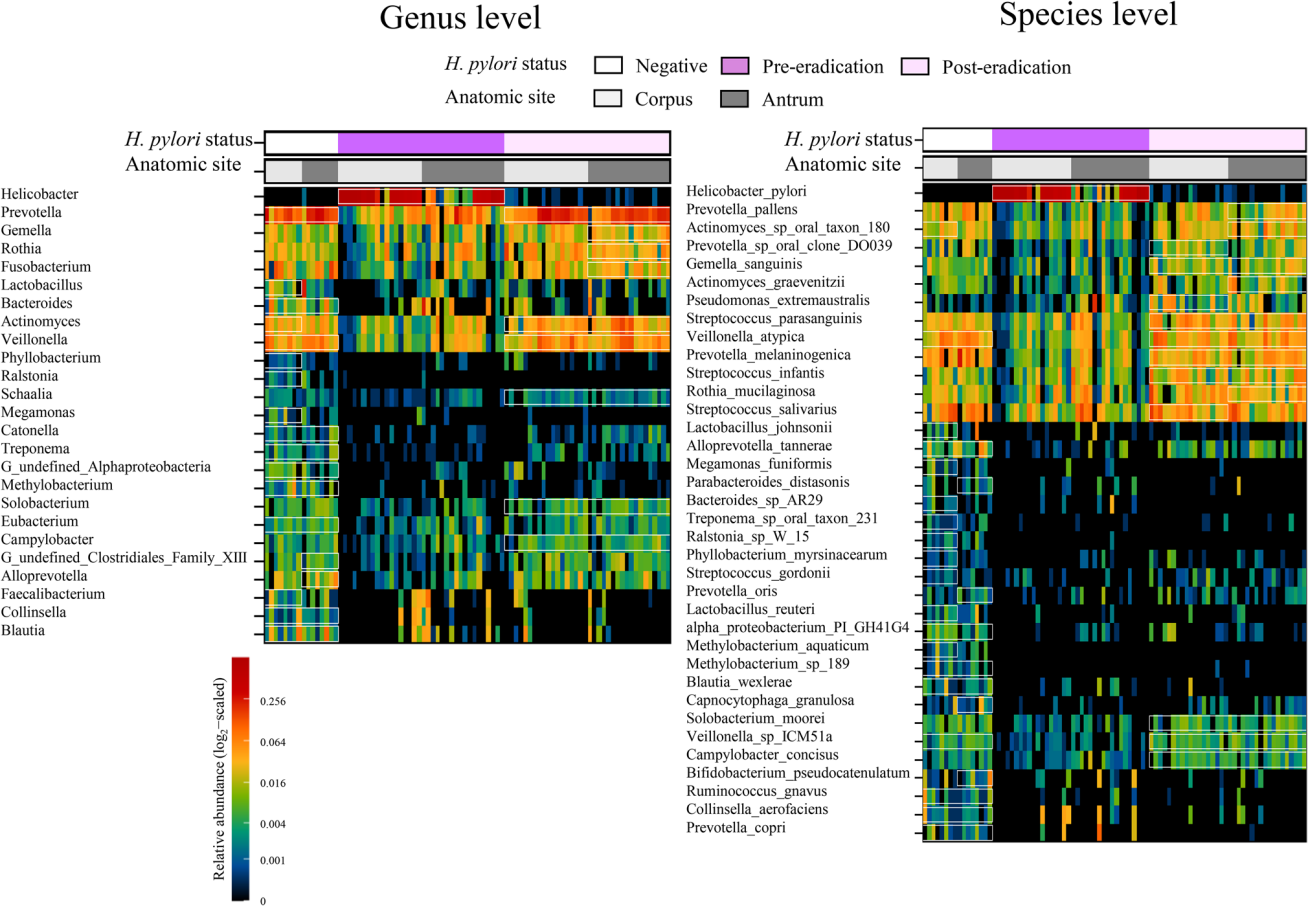
Heatmap of the relative abundance of gastric taxa stratified by *H. pylori* status and gastric site. Wilcoxon rank sum test was used to compare the relative abundances of gastric taxa in *H. pylori*-negative patients with those in pre-eradication or post-eradication patients. The Wilcoxon signed-rank test was used to compare the difference in paired samples from pre- and post-*H. pylori* eradication groups

The heatmap showed the genera that were abundant in the *H. pylori*-negative, pre-eradication, and post-eradication samples (Fig. 5). As compared to the bacteria in the negative and pre-eradication groups, *Helicobacter* was the only genus that was enriched in the pre-eradication group, whereas numerous genera, including *Veillonella*, *Bacteroides, Prevotella*, *Lactobacillus*, *Blautia*, *Ralstonia*, *Faecalibacterium*, *Methylobacterium*, and *Megamonas,* were enriched in the *H. pylori*-negative patients (Supplemental Tables 5, 6). We also observed a significant enrichment in the abundance of five genera (*Blautia*, *Ralstonia*, *Faecalibacterium*, *Methylobacterium*, and *Megamonas*) in the *H. pylori-*negative patients as compared to the genera in the post-eradication group. *H. pylori* eradication markedly reduced the abundance of the genus *Helicobacter* and increased the abundance of several genera, including *Prevotella*, *Veillonella*, *Actinomyces*, and *Solobacterium* (Fig. 5, Supplemental Table 6). Furthermore, there were significant differences in the relative abundances of 8 and 23 species in the antrum and corpus, respectively, between *H. pylori*-negative and pre-eradication groups. All the taxa, excluding *H. pylori,* were enriched in the negative group (Fig. 5, Supplemental Tables 7, 8). As compared to the microbial population in the post-eradication group, we identified ten and fifteen species in the antrum and corpus, respectively, that were enriched in the negative group. Among these species, *Ralstonia* sp. W-15 was the most enriched in the corpus, with an average abundance of 4.66%. Successful eradication of *H. pylori* significantly altered the abundance of 12 species. As expected, this reduced the abundance of *H. pylori* from 25.295% to 0.033 and 61.148 to 0.044% in the antrum and corpus, respectively (Supplemental Table 7). The other species belonging to the genera *Streptococcus*, *Veillonella*, *Prevotella*, *Gemella*, *Pseudomonas*, *Solobacterium,* and *Campylobacte*r increased after the eradication of *H. pylori* (Fig. 5, Supplemental Table 8).

## Discussion

Previous studies have demonstrated that *H. pylori* infection is associated with marked changes in the gastric microbiota [14, 19, 22–24]. Here, we confirmed that *H. pylori*-negative and -positive patients had different bacterial profiles characterized by less richness of gastric microbiota and the reduction in abundances of *Blautia*, *Ralstonia*, *Faecalibacterium*, *Methylobacterium*, and *Megamonas* in *H. pylori*-positive patients. Furthermore, there were significant differences in the α- and β-diversities between these two patient groups even after removing the *H. pylori*-related OTUs, indicating that changes in the gastric microbiota in the *H. pylori-*positive patients were not merely due to the presence of *H. pylori*. Dysbiosis persisted for a long time after eradicating *H. pylori*.

Studies have reported the restoration of the richness and evenness of gastric microbiota to a status similar to that of the negative subjects by the eradication [22, 23]; however, we failed to demonstrate marked effects of *H. pylori* eradication on microbial diversity. Among the α-diversity metrices, the Shannon index accounts for richness and evenness of species, while the remaining three metrices represented richness only. If some species are much more abundant than others, the Shannon index is low, thereby accounting for the increase in the Shannon index observed upon the eradication of *H. pylori*. In contrast, our findings showed that the eradication of *H. pylori* did not affect the number of OTUs and Chao1 and ACE indices. Moreover, these metrices were lower in the eradicated subjects than those in the *H. pylori-*negative subjects, suggesting that *H. pylori* eradication may be less effective in improving the richness of gastric microbiota in patients with early GC. Li et al. reported that *H. pylori* eradication normalized the composition of the microbial community of the stomach [23]. Guo et al. [22] demonstrated a significant change in β-diversity after the eradication of *H. pylori*, as assessed using UniFrac analysis. However, the composition of the microbial community in the eradicated subjects was significantly different from that in *H. pylori*-negative patients. Furthermore, we found that the microbiome profiles differed between the antrum and corpus in the *H. pylori-*positive group, with less bacterial richness in the corpus, although this difference was not observed in other groups. These findings were also contrary to previous studies that reported that paired tissue samples from the antrum and corpus had similar bacterial composition and diversity, regardless of the presence of *H. pylori* [23, 25, 26]. This can be attributed to the differences in the methodologies used in published literature and this study. However, we believe that the strict patient enrollment protocol employed in this study has resulted in significant differences in the microbial population and richness according to *H. pylori* colonization status and gastric anatomic sites. In this study, only subjects who met all the five selection criteria were enrolled in the *H. pylori*-negative group; thus, this group comprised only *H. pylori*-naïve subjects. The majority of the published studies enrolled patients who were negative for the *H. pylori* antibody, rapid urease test, or UBT as part of the *H. pylori*-negative cohort. Therefore, this group may include patients after eradication or after spontaneous clearance of *H. pylori* as well as *H. pylori*-naïve individuals, suggesting the heterogeneity of the population. Furthermore, we only enrolled patients who underwent ESD for early GC in the *H. pylori*-positive group. Gastric microbiota changes during the progression of *H. pylori*-induced gastritis to atrophic gastritis, intestinal metaplasia, and GC [23, 27]. Wang et al. reported that the changing patterns of the composition and function of the gastric microbiota are highly indicative of the stages of neoplastic progression in the stomach [27]. Thus, the gastric microbiota and how *H. pylori* eradication affects these profiles in patients with different *H. pylori*-induced pathologies should be analyzed independently. Moreover, *H. pylori* prefers to colonize the normal gastric mucosa and gradually decreases in abundance during the progression of gastritis from the antrum to the corpus before finally disappearing. The average abundance of *H. pylori* in the antrum and corpus was 22.3% and 61.1%, respectively, in our patients, who exhibited moderate to severe atrophic gastritis. Since the α-diversity matrices were inversely correlated with the abundance of *H. pylori*, the differences in the diversity of the microbiota in our patients may be due to the predominance of *H. pylori* in the corpus. To the best of our knowledge, this is the first report on the incomplete reversal of gastric dysbiosis on the eradication of *H. pylori* in early GC patients who have a high risk for metachronous GC even after successful eradication [28]

Experimental studies have suggested that microbes other than *H. pylori* may also be involved in gastric carcinogenesis [11–13], although direct clinical evidence for a link between specific dysbiosis or bacterial taxa and the development of GC remains to be uncovered. Hsieh et al. reported an increased abundance of *Clostridium* and *Fusobacterium* in GC tissues [29]. Ferreira et al. demonstrated that the microbiota in GC tissues was characterized by the reduction in the abundance of *Helicobacte*r and over-representation of intestinal commensals, including *Citrobacter*, *Clostridium*, *Lactobacillus*, *Achromobacter,* and *Rhodococcus* [30]. In contrast, Coker et al. observed the significant enrichment of the oral microbiome, including *Parvimonas micra*, *Peptostreptococcus stomatis*, *Fusobacterium nucleatum,* and *Gemella* in GC tissues compared to their abundance in the precancerous tissues [31]. Thus, the data were discordant, but microbial populations show alterations, although not identical in all patients, in GC tissues. More importantly, several studies have shown that the paired tumor and non-tumor tissue samples had similar microbiota profiles [23, 27, 32], suggesting the importance of the dysbiosis of background mucosa in the GC development. Compared to the negative group of patients, only *Helicobacter* was significantly enriched, while more than ten taxa were significantly reduced in the pre-eradication group. Among these reduced taxa, *Blautia*, *Ralstonia*, *Faecalibacterium*, *Methylobacterium*, and *Megamonas* were also found to be more deleted in the post-eradication group as compared to the negative group. Therefore, the deletion of these taxa may be involved as a common mechanism for primary and metachronous GC after the eradication of *H. pylori*. Anti-inflammatory effects have been demonstrated for *Blautia* and *Faecalibacterium* in gastrointestinal diseases, including inflammatory bowel diseases and intestinal graft-versus-host disease [33–36]. Interestingly, colorectal cancer tissues show a reduction in the abundance of these taxa [37, 38]. Taken together, our data and that previously published suggest that reduced abundances of *Blautia* and *Faecalibacterium* may maintain or enhance inflammation in *H. pylori*-infected and -eradicated gastric mucosa, thereby leading to the development of GC. *Ralstonia*, a Gram-positive bacterium, is enriched in the gastric mucosa of patients with GC before subtotal gastrectomy [39] and those with gastric inflammation one year after eradication therapy [40]. Thus, the reduction of *Ralstonia* is unlikely to be linked to GC. The pathogenicity of *Methylobacterium* and *Megamonas* in the stomach has not been studied; however, they may not be crucial during gastric carcinogenesis since they are found at an average abundance of < 1%.

This study has some limitations. First, we used a small patient cohort. Larger sample sizes will help detect sensitive differences in the microbiota between groups. Our strict criteria for the enrollment of *H. pylori*-naïve and *H. pylori*-positive patients with early GC and analysis of bacteria using samples obtained from the same site in the stomach will reduce intra-group variation and minimize the bias associated with small cohorts. Second, we were unable to match the patients in *H. pylori-*negative and -positive patients based on age and sex. However, several studies have detected no association between gastric microbial features and patient age or sex [23, 26]. Thus, we believe this would not majorly affect this study. Third, we enrolled patients who underwent ESD for early GC since they were at high risk for developing GC even after successful eradication of *H. pylori*. However, during the follow-up period, no patient metachronously developed GC, suggesting the validation of the study findings in patients with metachronous GC. Finally, the follow-up period after eradication therapy was not the same for all the patients; a majority of the patients were followed up for > 12 months, with the shortest period being 8 months. The effects of the eradication of *H. pylori* on gastric microbiota were assessed after a sufficient interval.

In conclusion, gastric dysbiosis may persist for a long time after the successful eradication of *H. pylori* in patients with a history of GC. Alterations in the gastric microbiota may form the underlying mechanism involved in the development of metachronous GC after the eradication of *H. pylori* as well as primary GC.

**Table 1:** Baseline characteristics of patients

	*H. pylori*-negative group (*n* = 10)	Pre-eradication group (*n* = 19)	*p* value
Age (years)			
Median ( interquartile ranges)	63.0 (55.75–67.0)	67.0 (64.0–74.0)	0.107
Sex			
Male	7	17	
Female	3	2	0.306
Severity of endoscopic atrophy			
None or mild (C-1 or C-2)	10	0	
Moderate (C-3 or O-1)	0	11	
Severe (O-2 or O-3)	0	8	< 0.001
Endoscopic diagnosis			
Normal	5	0	
Reflux esophagitis	1	0	
Gastric ulcer scar	2	0	
Fundic gland polyp	2	0	
Early gastric cancer (past/present)	0 (0/0)	19 (14/5)	

## Electronic supplementary material

Below is the link to the electronic supplementary material.

Supplementary material 1 (PDF 416 kb)

Supplementary material 2 (XLSX 10 kb)

Supplementary material 3 (XLSX 334 kb)

Supplementary material 4 (XLSX 12 kb)

Supplementary material 5 (XLSX 12 kb)

Supplementary material 6 (XLSX 18 kb)

Supplementary material 7 (XLSX 14 kb)

Supplementary material 8 (XLSX 18 kb)

Supplementary material 9 (XLSX 17 kb)

Supplementary material 10 (DOCX 15 kb)
