# Supplementary material for: Long-term persistence of gastric dysbiosis after eradication of Helicobacter pylori in patients who underwent endoscopic submucosal dissection for early gastric cancer
Source: Gastric Cancer. 2020 Nov 17;24(3):710–20. doi: 10.1007/s10120-020-01141-w (PMC8065006; doi:10.1007/s10120-020-01141-w)
Supplement: Supplementary file 1 — Supplementary material 1 (PDF 416 kb) [file 10120_2020_1141_MOESM1_ESM.pdf]

**Supplementary Materials to:**

# Long-term persistence of gastric dysbiosis after eradication of *Helicobacter pylori* in patients who underwent endoscopic submucosal dissection for early gastric cancer

Toshio Watanabe*[1], Yuji Nadatani[1], Wataru Suda[2], Akira Higashimori[1], Koji Otani[1], Shusei Fukunaga[1], Shuhei Hosomi[1], Fumio Tanaka[1], Yasuaki Nagami[1], Koichi Taira[1], Tetsuya Tanigawa[1,3], Geicho Nakatsu[4], Masahira Hattori[2,5], Yasuhiro Fujiwara[1]

[1]Department of Gastroenterology, Osaka City University Graduate School of Medicine, Osaka, Japan
[2]Laboratory for Microbiome Sciences, Center for Integrative Medical Sciences, RIKEN, Yokohama, Kanagawa, Japan.
[3]Department of Gastroenterology, Osaka City Juso Hospital, Osaka, Japan
[4]Department of Immunology and Infectious Diseases/Genetics and Complex Diseases, Harvard T. H. Chan School of Public Health, Boston, Massachusetts, United States.
[5]Graduate School of Advanced Science and Engineering, Waseda University, Tokyo, Japan.

**Corresponding Author:** Toshio Watanabe, MD, PhD
Department of Gastroenterology, Osaka City University Graduate School of Medicine
Postal address: 1-4-3 Asahi-machi Abeno-ku, Osaka City, Japan
Telephone number: +81-6-6645-3811
Fax number: +81-6-6645-3813
Email address: watanabet@med.osaka-cu.ac.jp

**Short running head:** Dysbiosis after eradication of *H. pylori*

Supplemental Figure 1

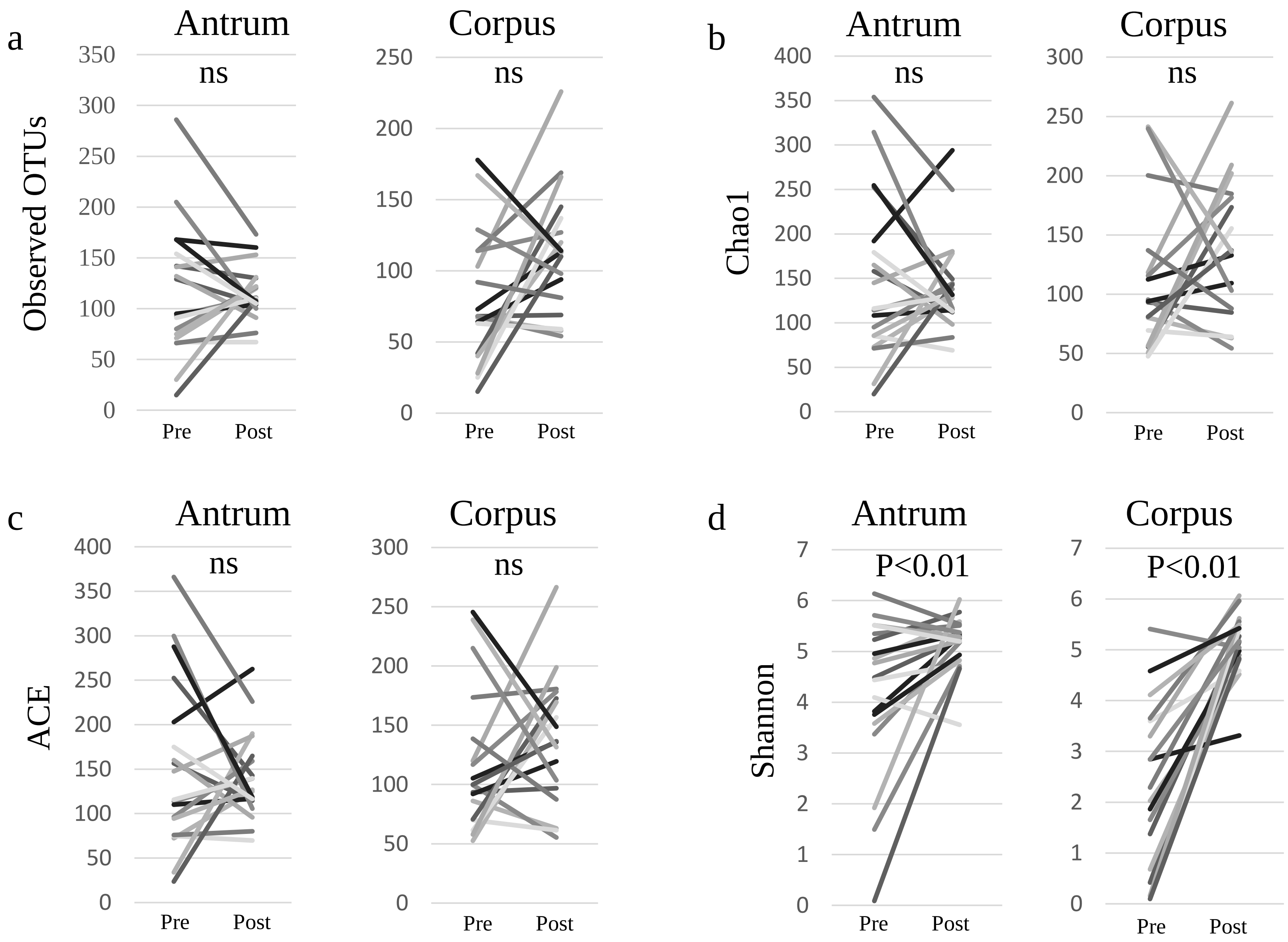

Supplemental Figure 2

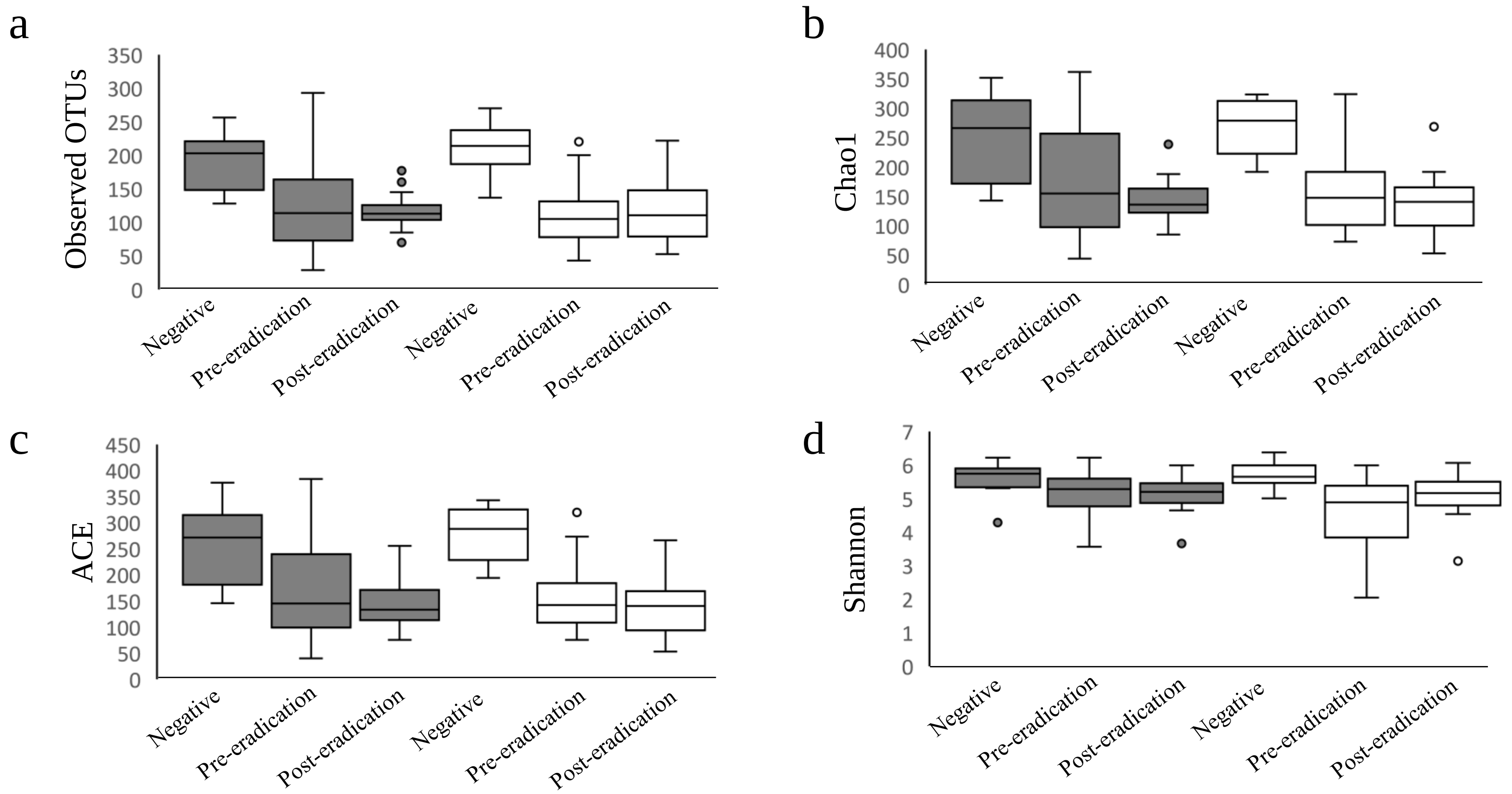


e

| Group comparison | Observed OTUs | Chao1 | ACE | Shannon |
|---|---|---|---|---|
| Antrum vs. corpus | | | | |
| Negative | 0.945 | 0.945 | 0.945 | 0..641 |
| Pre-eradication | 0.433 | 0.734 | 0.671 | 0.196 |
| Post-eradication | 0.257 | 0.212 | 0.167 | 0.899 |
| Negative vs. pre-eradication | | | | |
| Antrum | 0.007 | 0.054 | 0.016 | 0.028 |
| Corpus | <0.001 | 0.002 | <0.001 | <0.001 |
| Negative vs. post-eradication | | | | |
| Antrum | <0.001 | <0.001 | <0.001 | 0.028 |
| Corpus | <0.001 | <0.001 | <0.001 | 0.005 |
| Pre-eradication vs. Post-eradication | | | | |
| Antrum | 0.515 | 0.374 | 0.515 | 0.983 |
| Corpus | 0.966 | 0.347 | 0.369 | 0.024 |

Supplemental Figure 3

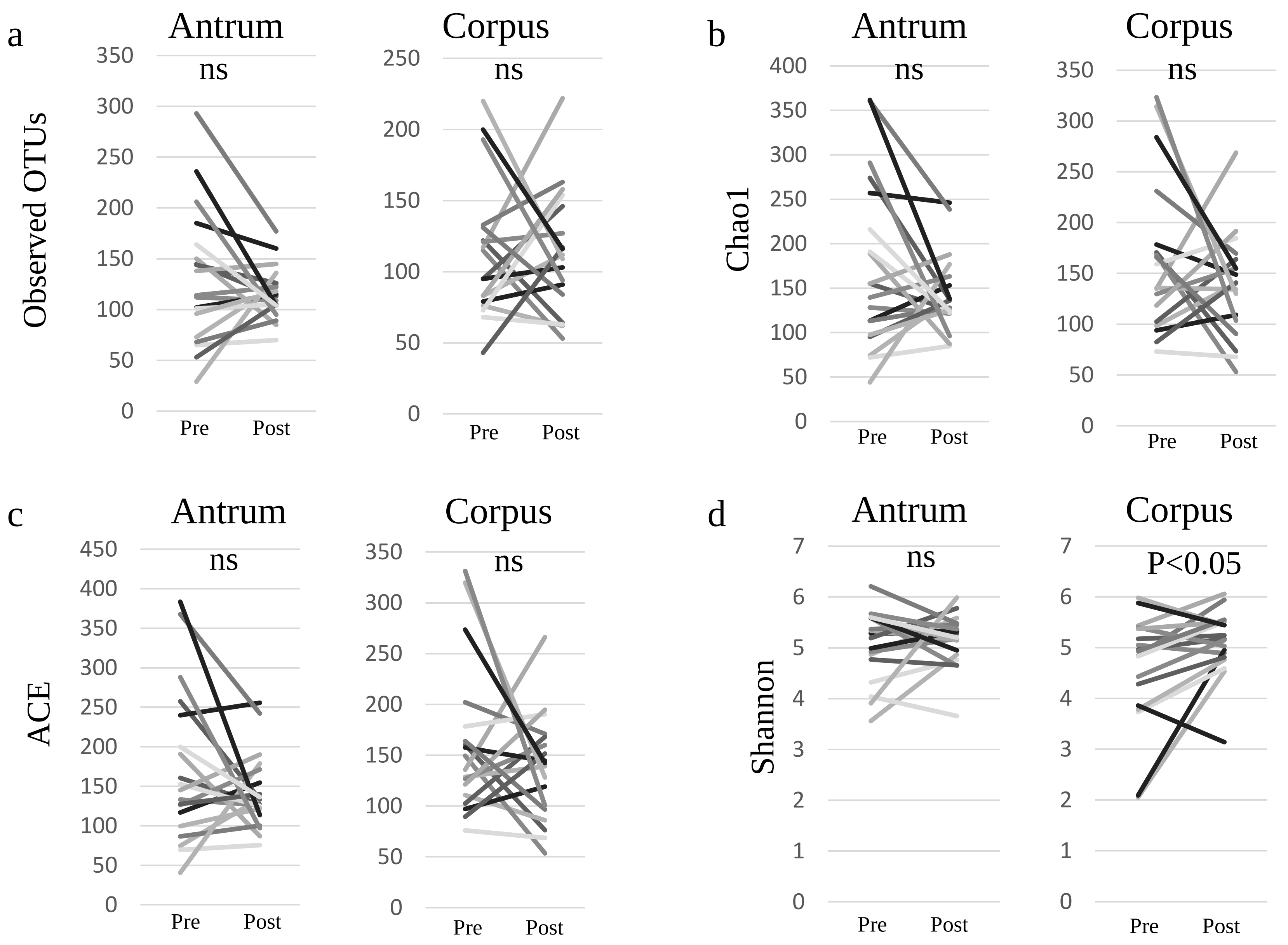

Supplemental Figure 4

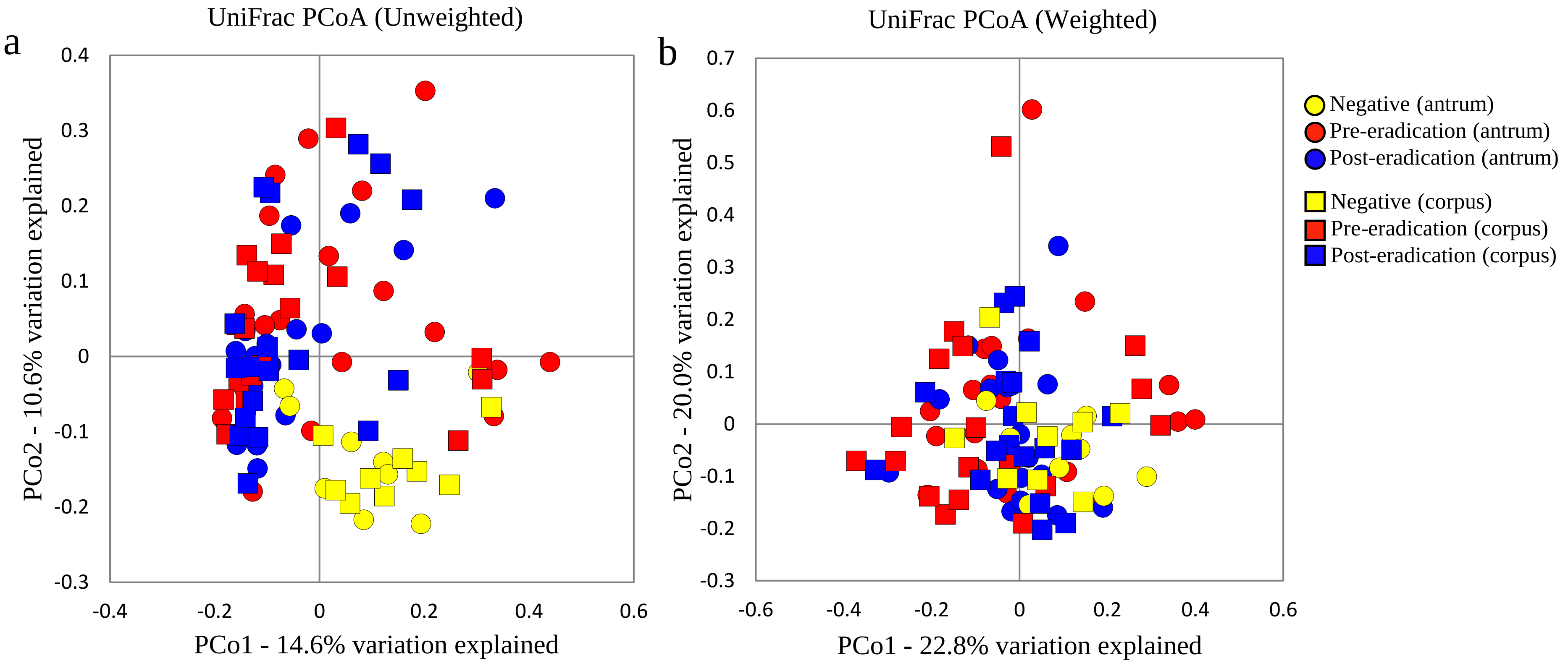


c

| Group comparison | Unweighted UniFrac | | Weighted UniFrac | |
|---|---|---|---|---|
| | $R^2$ | *P* value | $R^2$ | *P* value |
| Antrum vs. corpus | | | | |
| Pre-eradication | 0.03 | 0.287 | 0.034 | 0.269 |
| Negative vs. pre-eradication | | | | |
| Antrum | 0.074 | <0.007 | 0.089 | 0.029 |
| Corpus | 0.152 | <0.001 | 0.055 | 0.187 |
| Negative vs. post-eradication | | | | |
| Antrum | 0.084 | <0.002 | 0.097 | 0.013 |
| Corpus | 0.130 | <0.001 | 0.055 | 0.204 |
| Pre-eradication vs. Post-eradication | | | | |
| Antrum | 0.040 | 0.057 | 0.038 | 0.179 |
| Corpus | 0.032 | 0.239 | 0.060 | 0.049 |

## Supplemental figure legends

**Supplemental Figure 1. Effect of *H. pylori* eradication on α-diversity of the gastric microbiome.** The Wilcoxon signed-rank test was used to compare the difference in paired samples in the pre- and post-*H. pylori* eradication groups. (a) the numbers of observed OTUs. (b-d) indices of Chao1 (b), ACE (c), and Shannon (d).

**Supplemental Figure 2. Comparison of α-diversity of the gastric microbiome between different gastric regions or *H. pylori* status upon removing *H. pylori* reads.** The numbers of observed OTUs (a) and indices of Chao1 (b), ACE (c), and Shannon (d) in each group are expressed as medians and interquartile ranges. (E) *P*-values between different groups are shown. The Wilcoxon rank sum test was used to compare the values of the *H. pylori*-negative group with those of the pre-eradication or post-eradication patients. The Wilcoxon signed-rank test was used to compare the difference in paired samples of the pre- and post-*H. pylori* eradication groups.

**Supplemental Figure 3 . Effect of *H. pylori* eradication on α-diversity of the gastric microbiome (after removing *H. pylori* reads).** The Wilcoxon signed-rank test was used to compare the difference in paired samples from the pre- and post-*H. pylori* eradication groups. (a) the numbers of observed OTUs. (b-d) indices of Chao1 (b), ACE (c), and Shannon (d).

**Supplemental Figure 4. β-Diversity distances between different *H. pylori* status groups using PCoA (after removing *H. pylori* reads).** (a) Unweighted UniFrac PCoA (b) Weighted UniFrac PCoA. (c) PERMANOVA data comparing β-diversity of the gastric bacterial communities between different *H. pylori* status groups using unweighted and weighted UniFrac distances.
