# Supplementary material for: Long-term persistence of gastric dysbiosis after eradication of Helicobacter pylori in patients who underwent endoscopic submucosal dissection for early gastric cancer
Source: Gastric Cancer. 2020 Nov 17;24(3):710–20. doi: 10.1007/s10120-020-01141-w (PMC8065006; doi:10.1007/s10120-020-01141-w)
Supplement: Supplementary file 10 — Supplementary material 10 (DOCX 15 kb) [file 10120_2020_1141_MOESM10_ESM.docx]

**Supplementary Materials to:**

**Long-term persistence of gastric dysbiosis after eradication of *Helicobacter pylori* in patients** **who underwent** **endoscopic submucosal dissection for early gastric cancer**

Toshio Watanabe*^1^, Yuji Nadatani^1^, Wataru Suda^2^, Akira Higashimori^1^, Koji Otani^1^, Shusei Fukunaga^1^, Shuhei Hosomi^1^, Fumio Tanaka^1^, Yasuaki Nagami^1^, Koichi Taira^1^, Tetsuya Tanigawa^1,3^, Geicho Nakatsu^4^, Masahira Hattori^2,5^, Yasuhiro Fujiwara^1^

^1^Department of Gastroenterology, Osaka City University Graduate School of Medicine, Osaka, Japan

^2^RIKEN Center for Integrative Medical Sciences Laboratory for Microbiome Sciences , Yokohama, Kanagawa, Japan.

^3^Department of Gastroenterology, Osaka City Juso Hospital, Osaka, Japan

^4^Department of Immunology and Infectious Diseases/Genetics and Complex Diseases, Harvard T. H. Chan School of Public Health, Boston, Massachusetts, United States.

^5^Graduate School of Advanced Science and Engineering, Waseda University, Tokyo, Japan.

**Corresponding Author:** Toshio Watanabe, MD, PhD

Department of Gastroenterology, Osaka City University Graduate School of Medicine

Postal address: 1-4-3 Asahi-machi Abeno-ku, Osaka City, Japan

Telephone number: +81-6-6645-3811

Fax number: +81-6-6645-3813

Email address: [watanabet@med.osaka-cu.ac.jp](mailto:watanabet@med.osaka-cu.ac.jp)

**Short running head:** Dysbiosis after eradication of *H. pylori*

**Statistical analysis**

Results are expressed as medians and interquartile ranges for continuous variables. The Wilcoxon rank sum test was used to compare the continuous values in H. pylori-naïve patients (*H. pylori*-negative group) with those of *H. pylori*-positive patients with early GC (pre-eradication group) or after *H. pylori* eradication (post-eradication group), whereas categorical data were analyzed using Fisher’s exact test. The Wilcoxon signed-rank test was used to compare the differences between paired samples. The Spearman's rank method was used to analyze the correlation between groups. P<0.05 was considered statistically significant. All statistical analyses were performed using SPSS 21 for Windows (SPSS Inc.; Chicago, Illinois, United States) or R version 3.2.5 (R Foundation for Statistical Computing, Vienna, Austria).

α-diversity was evaluated using the number of observed OTUs and indices of Chao1, ACE, and Shannon. β-diversity (i.e., diversity among samples) was assessed using UniFrac distances. Weighted (i.e., quantitative) and unweighted (i.e., qualitative) UniFrac distances between groups were subjected to ADONIS similarity analysis. We performed PERMANOVA based on UniFrac distances to understand the differences between groups. Principal coordinate analysis plots were used to visualize differences in the microbial composition between groups. We used 0.10 as the cut-off for the false discovery rate to determine the significance in the differences in taxonomic abundance among groups.
